# Use of alcohol and drugs by employees in selected business areas in Norway: a study using oral fluid testing and questionnaires

**DOI:** 10.1186/s12995-015-0087-0

**Published:** 2015-12-16

**Authors:** Hilde Marie Erøy Edvardsen, Inger Synnøve Moan, Asbjørg S. Christophersen, Hallvard Gjerde

**Affiliations:** Norwegian Institute of Public Health, P. O. Box 4404, Nydalen, NO-0403 Oslo Norway; Norwegian Institute for Alcohol and Drug Research (SIRUS), P. O. Box 565, Sentrum, NO-0105 Oslo Norway

**Keywords:** Alcohol drinking, Illicit drugs, Prescription drugs, Workplace, Prevalence

## Abstract

**Background:**

Alcohol or drug use and associated hangover may reduce workplace safety and productivity and also cause sickness absence. The aims of this study were to examine (i) the use of alcohol and drugs, and (ii) reduced efficiency at work and absence due to such use among employees.

**Methods:**

Forty-four companies were invited; half of them agreed to participate. Employees filled in a questionnaire and provided a sample of oral fluid, which was analysed for alcohol, 12 psychoactive medicinal drugs and 6 illicit drugs. Participation was voluntary and anonymous.

**Results:**

Two thousand four hundred thirty-seven employees in eight business areas agreed to participate (92 % of those invited). By combining questionnaires and oral fluid testing, we found that 5.2 % had used psychoactive medication during the last couple of days, 1.4 % had used illicit drugs, 17.0 % had used alcohol during the last 24 h but only one person (0.04 %) was positive for alcohol in oral fluid. About 25 % reported reduced efficiency at work, and 5 % reported absence from work due to alcohol use during the past 12 months. The use of illicit drugs and binge drinking resulting in reduced efficiency and absence was most common among restaurant and bar workers and more common among men than women, whereas use of psychoactive medication was most common among healthcare, transportation and storage workers.

**Conclusion:**

Impairment at work due to alcohol or drugs was rare, whereas reduced efficiency due to drinking was reported by a fairly large proportion. There were marked differences between some business areas, and across gender.

## Background

Use of alcohol or drugs and associated hangover effects may reduce workplace safety and productivity [1–3] and also cause sickness absence [4, 5]. Little is known about the use of alcohol and drugs that may affect safety and efficacy at work in Norway. A study published in 2004 found that 4 % reported having been under the influence of alcohol or drugs at work and 4 % had been absent from work because of alcohol and drug use [6]. Of the participants, 2.6 % reported having used illegal drugs and 18 % prescribed drugs during the past 12 months.

While few studies have addressed the prevalence of both alcohol and drug use and the consequences of such use in a work setting, the alcohol use-sickness absence association have been addressed in a number of studies. A recent review of 28 studies applying individual-level survey data to study a total of 48 associations between various measures of alcohol use and sickness absence, showed that there is strong empirical evidence for an association between alcohol use and both short- and long-term sickness absence [7]. One of the studies included in that review was conducted among young employees in Norway, where 8.1 % reported that they had been absent from work due to alcohol use the past 12 months [8]. This study found that the proportion of young male employees who reported having alcohol-related sickness absence was nearly twofold that of women, i.e., 10.5 and 5.7 %, respectively. This finding is consistent with results in other studies [7]. However, less is known about gender differences with respect to drug use and consequences of such use in a workplace setting.

Results from workplace drug testing (WDT) in Norway for the period 2000–2006 showed that 2.9 % of the analysed samples were positive for drugs; only 1.0 % for illicit drugs [9]. However, WDT is used only within few business areas in Norway, primarily in shipping, oil industry and transportation, and it is likely that random workplace drug testing reduces the incidence of drug use among employees. Therefore, WDT findings do not accurately reflect the incidence of drug use among Norwegian employees in general.

The use of alcohol and drugs varies between countries and different business areas. American studies found that heavy alcohol use and illicit drug use was most prevalent among employees within the construction industry, arts, entertainment, recreation, mining, accommodation and food services and least prevalent among healthcare, social assistance and educational services [10, 11]. Differences between business areas have previously not been studied in Norway.

Research on the use of alcohol or drugs in relation to work has in most cases been performed using questionnaires or interviews. However, the use of alcohol and drugs is commonly under-reported [12–14]. A number of studies have found that analysis of biological samples may provide more accurate data than self-reports on alcohol and drug use during the last days or months [15–17]. However, drug testing cannot reveal alcohol and drug using habits and consequences of such use, so a combination of drug testing and questionnaires or interviews provides more data than using a single method alone [18–21].

The aims of this study were: (i) to examine the use of alcohol and drugs using a combination of self-report through questionnaires and testing of oral fluid (mixed saliva), and (ii) self-reported sickness absence and reduced efficiency or hangover at work due to such use among employees in eight business areas in Norway, and across gender.

## Methods

We first performed a pilot study that included 526 employees during 2008–2009 [22], and a follow-up study with 1911 employees was conducted during 2011–2014. We present the total findings from both studies in this article.

### Ethics

The study was approved by the Regional Committee for Medical and Health Research Ethics. The dataset was completely anonymous.

### Consent

Oral informed consent was obtained from the participants for publication of reports.

### Study design and setting

The recruitment of companies was performed during 2008–2013. Forty-four companies and business chains were invited to participate, and a general call for participation was published in magazines and on websites. In total, 21 companies agreed to participate, and the Norwegian Public Roads Administration agreed to let us recruit truck drivers at control stations for heavy traffic; thus, altogether 22 businesses participated.

Information about the upcoming study was distributed to all employees except truck drivers several weeks before the recruitment of employees was performed. The date for recruitment was not announced. First, the study days were selected for each company; then, either a random selection or all employees who were present in the building were contacted. Occupational drivers were recruited at a heavy vehicle checking station during scheduled controls.

For all companies except one, and for all occupational drivers, each employee was approached individually by one project assistant from the Norwegian Institute of Public Health (NIPH) and asked to participate. Written and verbal information about the project was given, and oral informed consent was obtained from all participants. Those who agreed to participate filled in a questionnaire in an area shielded from view and provided an oral fluid specimen. The questionnaire and the sample of oral fluid were placed in an unlabelled envelope that was closed and sealed and collected by a project assistant within approximately an hour.

For one company, an envelope containing the questionnaire and sampling device for oral fluid, including instructions for use, was given to random employees when entering the company facilities in the morning. The employees were asked to deliver the questionnaire and the oral fluid sample in closed and sealed, unlabelled envelopes at specified sites before noon.

The recruitment of employees was completed in 2014. In total, 2639 employees were invited and 2437 agreed to participate (92 %). The included business areas were healthcare (917 employees), finance (457 employees), manufacturing (254 employees), transportation/storage (233 employees), restaurants/bars (131 employees), public administration (211 employees), media (152 employees; questionnaires only), and research institutes (82 employees). Participation rates and socio-demographic characteristics for business areas with two or more companies and more than 100 participating employees are presented in Table 1. Data for employees in public administration, media and research are presented in the column named “Other”.

**Table 1:** Employee participation rates, age and gender of all participants (*N* = 2437) across business areas

	Healthcare	Finance	Industry	Transportation and storage	Restaurants and bars	Other	Total
No. of participants	917	457	254	233	131	445	2437
Participation rate, %	98.3	96.4	91.0	95.9	92.9	78.2	92.3
Gender, % (n)							
Women	79.5 (729)	47.9 (219)	19.3 (49)	6.4 (15)	47.3 (62)	54.6 (243)	54.0 (1317)
Men	18.5 (170)	49.0 (224)	77.2 (196)	77.7 (181)	51.1 (67)	38.9 (173)	41.5 (1011)
Not reported	2.0 (18)	3.1 (14)	3.5 (9)	15.9 (37)	1.5 (2)	6.5 (29)	4.5 (109)
Age distribution, % (n)							
<30 years	14.7 (135)	9.4 (43)	11.8 (30)	23.2 (54)	78.6 (103)	14.6 (65)	17.6 (430)
30–39 years	26.8 (246)	28.2 (129)	15.0 (38)	18.0 (42)	16.8 (22)	28.1 (125)	24.7 (602)
40–49 years	26.7 (245)	24.7 (113)	30.7 (78)	22.7 (53)	3.1 (4)	22.9 (102)	24.4 (595)
50–59 years	22.0 (202)	28.0 (128)	28.7 (73)	21.5 (50)	0.0 (0)	21.6 (96)	22.8 (549)
60+ years	8.2 (75)	8.5 (39)	11.0 (28)	7.3 (17)	0.8 (1)	11.0 (49)	8.6 (209)
Not reported	1.5 (14)	1.1 (5)	2.8 (7)	7.3 (17)	0.8 (1)	1.8 (8)	2.1 (52)

### Data collection

For most of the companies, the data collection was performed during weekdays only. Oral fluid was collected using Statsure Saliva Sampler™ (Statsure Diagnostic Systems, Framingham MA, USA). The time required for sample collection and filling in the questionnaire was about 5 min.

The samples of oral fluid were frozen within one day after collection and thawed once before the analysis. Alcohol was analysed by an automated enzymatic method [23]. Medicinal and illicit drugs were analysed by liquid chromatography-tandem mass spectroscopy; two similar analytical methods were used during the project period [24, 25]. The analysed compounds and cut-off concentrations (above which a sample was regarded as positive) are presented in Table 2.

**Table 2:** Substances analysed in oral fluid, cut-off concentrations and prevalence above cut-off concentrations

Compound	Description	Cut-off^c^ ng/mL	Prevalence % (n)
6-Acetylmorphine	Metabolite of heroin	1.3	0.0 (0)
Alcohol		0.10 g/L	0.04 (1)
Alprazolam	Benzodiazepine; anxiolytic	0.62	0.0 (0)
7-Aminoclonazepam	Metabolite of clonazepam	0.71	0.0 (0)
7-Aminoflunitrazepam	Metabolite of flunitrazepam	0.17	0.0 (0)
7-Aminonitrazepam	Metabolite of nitrazepam	0.63	0.0 (0)
Amphetamine	Stimulant^a^	24	0.09 (2)
Benzoylecgonine	Metabolite of cocaine	9.8	0.04 (1)
Clonazepam	Benzodiazepine; anticonvulsant, anxiolytic	0.63	0.0 (0)
Cocaine	Stimulant^b^	1.8	0.04 (1)
Codeine	Opioid analgesic, antitussive	7.5	0.3 (6)
Diazepam	Benzodiazepine; anxiolytic, anticonvulsant, sedative	0.40	0.7 (16)
Flunitrazepam	Benzodiazepine; anxiolytic	0.31	0.0 (0)
3,4-Methylenedioxy-methamphetamine (MDMA)	Psychedelic hallucinogenic drug^b^	26	0.0 (0)
Methadone	Opioid used mainly for opioid dependence, but also for analgesia	11	0.0 (0)
Methamphetamine	Stimulant^b^	15	0.1 (3)
Morphine	Opioid analgesic, also metabolite of codeine and heroin	7.1	0.2 (4)
Nitrazepam	Benzodiazepine; anxiolytic	0.56	0.0 (0)
Nordiazepam	Metabolite of diazepam	0.68	0.3 (6)
Oxazepam	Benzodiazepine; anxiolytic, anticonvulsant, and metabolite of diazepam	4.9	0.04 (1)
∆9-Tetrahydrocannabinol (THC)	Cannabis^a^	0.63	0.7 (16)
Zolpidem	Short acting hypnotic	1.2	0.09 (2)
Zopiclone	Short acting hypnotic	1.6	1.9 (43)

^a^Mostly used illegally in Norway

^b^Illegal in Norway

^c^Concentrations in neat oral fluid above which the analytical findings were regarded as positive

Two versions of the questionnaire were used. The questionnaire used for the pilot study in five businesses within transportation/storage, public administration, media and research did not include questions on drug use during the last 12 months; this question was added based on a request from one of the participating companies and was used for 17 businesses included after the pilot study had been finished.

### Statistical methods

Possible differences between the prevalence of medicinal or illicit drugs in oral fluid samples from different business areas were initially assessed using Pearson’s chi-square test for categorical data.

Adjusted odds ratios (OR) with 95 % confidence intervals (95 % CI) were calculated using multivariate unconditional logistic regression using SPSS Statistics Version 22 (IBM Corporation, Armonk, NY). Drug findings or self-reported data were included as dependent variable (with 2 categories; 0 = negative; 1 = positive). Independent variables were gender, age group (5 categories) and business areas (6 categories).

## Results

The participation rate among invited businesses was 50.0 %. Among the employees in the participating businesses the average participation rate was 92.3 %; when using the regular recruitment procedure 95.6 % (range 80.0–100.0 %) and 67.8 % when the participation when employees were asked to deliver the envelope with questionnaire and oral fluid sample on specified sites.

### Oral fluid

Positive alcohol and drug findings are presented in Table 2 and summarized in relation to business area and gender in Table 3. Only one employee (0.04 %) was positive for alcohol; this might be due to alcohol drinking the day before or due to a small alcohol intake during the working day, e.g. at lunch. Few employees were positive for illicit drugs (0.9 %) compared to medicinal drugs (3.0 %). The most frequently detected substances were the sleeping agent zopiclone (1.9 %), the sedative diazepam (0.7 %), cannabis (0.7 %), the analgesic substance codeine (0.3 %) and methamphetamine (0.1 %). The medicinal drugs that were found can in most cases be detected in oral fluid for more than 12 h after using a single dose, perhaps more than 24 h in some cases; amphetamine, cannabis (THC) and the cocaine metabolite benzoylecgonine may also be detected for more than 12 h, rarely longer than 48 h, whereas cocaine can be detected for less than 12 h after use [26].

**Table 3:** Self-reported alcohol and drug use and results from testing of oral fluid samples

	Healthcare	Finance	Manufacturing	Transportation and storage	Restaurants and bars	Other	Men	Women	Total
Oral fluid samples, % (n)	98.8 (906)	98.2 (449)	99.2 (252)	99.6 (232)	99.2 (130)	65.6 (292)	unk.^a^ (928)	unk.^a^ (1226)	92.8 (2261)
Questionnaires, % (n)	100.0 (917)	100.0 (457)	100.0 (254)	97.0 (226)	100.0 (131)	100.0 (445)	unk.^a^ (1011)	unk.^a^ (1317)	99.7 (2430)
Psychoactive medication, % (n)									
A: Detected in oral fluid	4.6 (42)	1.1 (5)	2.0 (5)	3.0 (7)	2.3 (3)	1.7 (5)	2.3 (21)	3.7 (45)	3.0 (67)
B: Self-reported use last 48 h	4.5 (41)	3.7 (17)	3.5 (9)	5.3 (12)	3.8 (5)	3.8 (17)	4.3 (43)	3.9 (52)	4.2 (101)
Either A or B	6.0 (55)	4.2 (19)	4.7 (12)	7.3 (17)	5.3 (7)	3.8 (17)	5.1 (52)	5.2 (69)	5.2 (127)
Self-reported non-therapeutic use last 12 months	0.7 (6)	0.7 (3)	1.2 (3)	nc	1.5 (2)	nc	nc	nc	nc
Illicit drugs, % (n)									
A: Detected in oral fluid	0.0 (0)	0.4 (2)	0.8 (2)	3.0 (7)	6.9 (9)	0.0 (0)	1.7 (16)	0.3 (4)	0.9 (20)
B: Self-reported last 48 h	0.0 (0)	1.1 (5)	0.0 (0)	0.9 (2)	11.5 (15)	0.0 (0)	1.3 (13)	0.7 (9)	0.9 (22)
Either A or B	0.0 (0)	1.3 (6)	0.8 (2)	3.9 (9)	12.2 (16)	0.0 (0)	2.3 (23)	0.8 (10)	1.4 (33)
Self-reported use last 12 m	1.9 (17)	3.7 (17)	1.6 (4)	nc	28.2 (37)	nc	nc	nc	nc
Alcohol, % (n)									
A: Detected in oral fluid	0.0 (0)	0.2 (1)	0.0 (0)	0.0 (0)	0.0 (0)	0.0 (0)	0.1 (1)	0.0 (0)	0.0 (1)
B: Self-reported use last 24 h	13.4 (123)	12.3 (56)	9.8 (25)	12.4 (28)	45.0 (59)	27.2 (121)	19.2 (194)	15.3 (201)	17.0 (412)
Either A or B	13.4 (123)	12.5 (57)	9.8 (25)	12.4 (28)	45.0 (59)	27.2 (121)	19.3 (195)	15.3 (201)	17.0 (413)
Drinking habits and consequences, % (n)									
Binge drinking (≥6 units) at least once a month	11.8 (108)	25.6 (117)	23.6 (60)	22.6 (51)	58.0 (76)	27.0 (120)	30.4 (307)	15.7 (207)	21.9 (532)
Reduced efficiency or hangover at work during previous 12 m	12.2 (112)	39.8 (182)	5.5 (14)	9.7 (22)	65.6 (86)	40.9 (182)	27.8 (281)	22.4 (295)	24.6 (598)
Absence from work due to drinking during previous 12 m	0.9 (8)	9.4 (43)	1.2 (3)	3.5 (8)	20.6 (27)	8.8 (39)	7.2 (73)	3.9 (51)	5.3 (128)

^a^Gender was not reported by 109 participants

nc: Data was not collected for all groups of participants

Medicinal drugs were detected more frequently (*p* <0.001) and illicit drugs less frequently (*p* <0.001) in samples of oral fluid from healthcare workers than in samples from employees in other business areas.

Illicit drugs were detected more frequently (*p* <0.001) in samples from restaurant/bar workers than in samples from employees in other business areas.

Of the 16 employees who were found to be positive for cannabis, the THC concentrations were above 2 ng/mL in native oral fluid (calculated using sample weight) in 14 cases; this concentration has been proposed as limit in the USA when oral fluid samples are used in workplace drug testing [27]. Four had concentrations between 25 and 300 ng/mL, suggesting cannabis smoking within a few hours before sampling. For two of the three employees who were positive for methamphetamine, concentrations were higher than the proposed limit of 50 ng/mL in the USA [27]. The concentrations were more than 1000 ng/mL, suggesting intake of moderate doses within the last 24 h or large doses 1–3 days ago. Those two individuals had combined methamphetamine with diazepam, which is a commonly used drug combination among problematic drug users. For five of the 43 employees who were positive for the sleeping agent zopiclone, the concentrations were above 50 ng/mL. Those high concentrations suggest that the medication might have been taken less than 6 h before sample collection. One of the diazepam users had 34 ng/mL in oral fluid, which indicates very recent drug intake or high concentration in blood. The other drug findings were of low concentrations that were unlikely to affect safety and efficacy.

### Questionnaires

The results from the questionnaire are presented in Table 3. Self-reported use of psychoactive medication was fairly similar across business areas (ranging from 3.5 to 5.3 %). However, large differences were observed for self-reported use of illicit drugs. The proportion who reported using illicit drugs during the last 48 h among restaurant/bar workers was 11.5 % compared to 1 % or less in other business areas.

Employees in four business areas were asked about the use of illicit drugs and the non-therapeutic use of psychoactive medication (recreational use to get intoxicated or high) during last 12 months. More than 25 % of the restaurant/bar workers reported illicit drug use, less than 4 % in other business areas. Less than 2 % reported recreational use of psychoactive medication.

Self-reported alcohol use during the last 24 h, binge drinking, reduced efficiency or hangover and absence from work due to drinking was also most frequently reported by restaurant/bar workers, and was also fairly frequently reported by finance workers and workers in the group called “Other”, which included media, research and public administration employees.

### Comparing oral fluid and questionnaires

Some under-reporting of drug use was observed when comparing drug findings in oral fluid and self-reported drug use. Of those who were positive for illicit drugs in oral fluid (*n* = 20), 45.0 % reported using illicit drugs during the last 48 h; whereas among those who were positive for medicinal drugs (*n* = 67), 61.2 % reported intake during the last 48 h.

When adding drug findings in oral fluid to self-reported drug used during the last 48 h, the number of medicinal drug users increased by about a quarter and the number of illicit drug users increased by about the half when compared to self-reported use only. When including either analytical findings or self-reported use, recent use of psychoactive medication was most prevalent among transportation and storage workers, whereas recent use of illicit drugs was most prevalent among restaurant/bar workers.

### Differences across business areas

The data presented in Table 3 suggest that there were marked differences between business areas. However, there were also significant differences between genders and age groups, making evaluation of the prevalence data more complicated. In order to study differences between business areas while adjusting for differences in the distributions of age and genders, we performed logistic regression analysis using drug findings or self-reported data as dependent variable, and business area, age and gender as covariates. The regression analyses for the detection of medicinal or illicit drugs in samples of oral fluid and self-reported inefficiency or absence due to drinking are presented in Table 4.

**Table 4:** Logistic regression analysis

	Univariate analysis			Multivariate analysis		
	OR	95 % CI	*p*	OR	95 % CI	*p*
Detection of illicit drugs in oral fluid						
Non-restaurant/bar employees (referent)						
Restaurant/bar	13.70	5.57–33.69	<0.001	5.00	1.82–13.72	0.002
Females	0.19	0.06–0.57	0.003	0.19	0.06–0.59	0.004
Age < 30 years (referent)						
Age 30–39 years	0.51	0.20–1.28	0.151	0.77	0.28–2.15	0.622
Age 40+ years	0.03	0.00–0.21	0.001	0.05	0.01–0.43	0.006
Detection of medicinal drugs in oral fluid						
Healthcare employees (referent)						
Finance	0.23	0.09–0.59	0.002	0.24	0.09–0.63	0.003
Manufacturing	0.34	0.12–0.95	0.040	0.35	0.11–1.05	0.062
Transportation/storage	0.76	0.34–1.73	0.516	0.92	0.35–2.41	0.872
Restaurant/bar	0.48	0.15–1.57	0.224	0.77	0.20–2.93	0.700
Other lines of business	0.39	0.15–1.00	0.050	0.39	0.15–1.00	0.050
Females	1.66	0.98–2.81	0.058	1.27	0.67–2.42	0.462
Age < 30 years (referent)						
Age 30–39 years	0.71	0.28–1.79	0.464	0.79	0.29–2.11	0.632
Age 40–49 years	1.32	0.58–2.98	0.512	1.50	0.61–3.68	0.375
Age 50–59 years	1.83	0.83–4.05	0.134	2.18	0.91–5.22	0.082
Age 60+ years	2.37	0.95–5.94	0.066	2.83	1.05–7.63	0.040
Reported reduced efficiency or hangover at work last 12 months due to drinking						
Healthcare employees (referent)						
Finance	4.82	3.66–6.35	<0.001	5.10	3.79–6.87	<0.001
Manufacturing	0.37	0.20–0.68	0.001	0.31	0.16–0.58	<0.001
Transportation/storage	0.74	0.44–1.25	0.265	0.45	0.26–0.79	0.006
Restaurant/bar	14.18	9.35–21.52	<0.001	5.75	3.66–9.05	<0.001
Other lines of business	4.85	3.67–6.42	<0.001	4.96	3.69–6.67	<0.001
Females	0.75	0.62–0.90	0.003	0.63	0.50–0.79	<0.001
Age < 30 years (referent)						
Age 30–39 years	0.47	0.36–0.61	<0.001	0.41	0.30–0.56	<0.001
Age 40–49 years	0.30	0.23–0.40	<0.001	0.31	0.22–0.43	<0.001
Age 50–59 years	0.20	0.15–0.27	<0.001	0.18	0.13–0.26	<0.001
Age 60+ years	0.16	0.10–0.26	<0.001	0.13	0.08–0.22	<0.001
Reported sickness absence last 12 months due to drinking						
Healthcare employees (referent)						
Finance	11.93	5.55–25.60	<0.001	11.04	5.06–24.07	<0.001
Manufacturing	0.92	0.19–4.36	0.916	0.80	0.17–3.86	0.778
Transportation/storage	4.22	1.51–11.79	0.006	2.73	0.94–7.97	0.066
Restaurant/bar	29.22	12.93–66.02	<0.001	13.76	5.78–32.77	<0.001
Other lines of business	10.92	5.04–23.67	<0.001	9.92	4.54–21.69	<0.001
Females	0.52	0.36–0.75	0.001	0.59	0.39–0.88	0.010
Age < 30 years (referent)						
Age 30–39 years	0.68	0.44–1.05	0.080	0.75	0.45–1.24	0.259
Age 40–49 years	0.23	0.13–0.42	<0.001	0.31	0.16–0.59	<0.001
Age 50–59 years	0.22	0.12–0.41	<0.001	0.28	0.14–0.55	<0.001
Age 60+ years	0.13	0.04–0.42	0.001	0.14	0.04–0.48	0.002

Due to the fact that illicit drugs were detected among employees in only some business areas and only one person above 40 years of age, the restaurant business were compared with non-restaurant business employees in total, and the employees were disaggregated into three age groups. Employees in the restaurant business had high odds ratio for being positive for illicit drugs (*p* = 0.002) when adjusting for gender and age group, compared to employees in other business areas.

Employees within the finance business and the group of businesses called “other” had significantly lower odds for being positive for medicinal drugs than healthcare employees. If comparing healthcare workers with employees within non-healthcare businesses in total, they were found to have higher odds ratios for being positive for medicinal drugs (*p* = 0.001) compared to other business areas (results not shown).

When compared with employees in the healthcare business, employees in the finance, restaurant and “other” businesses had significantly higher odds ratios for reporting reduced efficiency or hangover at work at least once during the previous 12 months (*p* <0.001) and sickness absence (*p* <0.001) due to drinking. Highest odds ratios were found for restaurant/bar workers.

### Gender differences

The results presented in Table 3 show that illicit drugs were detected more frequently among male employees than females, and self-reported binge drinking, reduced efficiency or hangover at work and sickness absence from work due to drinking was also more frequent among males.

Logistic regression analysis was performed adjusting for age group and the six business areas (Table 4). Female employees had statistically significantly lower odds ratios compared to men to report reduced efficiency or hangover at work during previous 12 months (*p* <0.001) and absence from work due to drinking during previous 12 months (*p =* 0.010). The difference observed between the genders for the detection of medicinal drugs in samples of oral fluid when adjusting for age group and business area were not statistically significant. However, the odds ratio for detection of illicit drugs was statistically significantly lower for females than males when adjusting for age group and business area when disaggregated into restaurant and non-restaurant businesses (*p* = 0.004).

## Discussion

In this study, we combined the use of questionnaires and oral fluid testing to compare alcohol and drug use, as well as sickness absence and reduced efficiency due to such use, across eight business areas in Norway, and across gender.

More detailed results from one business area (healthcare) have been published in an separate article [28]. In this article we present the total findings from the pilot and main studies, which included 2437 employees from eight business areas. Data from the pilot study are included to enable the comparison between all included business areas; this has not been reported for the pilot study previously.

Few employees were impaired by alcohol or drugs at the time of collection of oral fluid. One employee had concentration of alcohol of about 0.2 g/L in oral fluid (the concentration in blood is about the same as in oral fluid), which either may be caused by drinking one glass of beer or wine within the last hour, or residual alcohol after heavy drinking the day before. For drugs, it is impossible to accurately estimate concentrations in blood based on concentrations in oral fluid due to large individual variation [29]. However, about 10 persons (0.4 %) had drug concentrations in oral fluid that might be associated with recent drug use that may affect the performance at work.

In a U. S. survey, 8.1 % reported workplace use of alcohol during the last 12 months, 0.99 % reported weekly use, 0.78 % reported alcohol-related impairment weekly, and 9.23 % reported being hungover at work during the last 12 months [30]. In Europe, the situation varies a lot between different countries; in some countries the use of alcohol during the working day has been very common. The proportion of workers who consumed alcohol during the working day was reportedly 11 % in Austria, 14 % in Denmark, 8.2 % in Poland; whereas in the Netherlands, 4 % of the workers who drank alcohol sometimes drank before going to work or at work [31]. Thus, the use of alcohol in relation to work seemed to be very much less common in our study than in some European countries.

In a study based on data from a U.S. National Survey performed in 2002–2003, a total of 14.1 % of the workforce reported having used illicit drugs during the last 12 months, 3.6 % at least once a week, and 1.25 % reported use 6–7 days a week [32]. Thus, the use of illicit drugs was significantly more common among employees in the USA than in Norway. There is little information about drug use in relation to work for other European countries [31]. However, there is large variation in the use of illicit drugs in general between European countries [33].

The results show large differences between some business areas. Restaurant/bar workers reported more often alcohol use during the last 24 h. This is partly due to the fact that for those businesses the data collection included weekends, when approximately 70 % of drinking situations occur [34]. A larger proportion of restaurant/bar workers also reported binge drinking during the past 12 months compared to employees in other business areas as well as being less efficient at work and absence from work due to alcohol use during the last 12 months. Drug use during the last 48 h was also more common among restaurant/bar workers.

The findings among restaurant/bar employees are partly related to the large proportion of employees below 30 years of age, who are expected to use more alcohol and illicit drugs than older age groups. However, when adjusting for age and gender distributions, significantly more drug use and alcohol-related hangover and absence were found among restaurant/bar employees than among employees in most other business areas. Also the fact that they are working in an environment with high availability of alcohol and work-related norms that are supportive of after-work drinking and hangover at work may have influenced their drinking habits [35].

Also previous studies have found high alcohol consumption or high prevalence of hazardous drinking among restaurant workers, both in Scandinavia and elsewhere [10, 35–38]. Previous American studies have also found that restaurant workers more often reported use of illicit drugs than employees in many other business areas [10, 11].

The proportion of workers within finance and the “Other” category (i.e., media, research and public administration employees) who reported reduced efficiency and sickness absence due to alcohol was also fairly high.

It has previously been reported that problematic alcohol and drug use may be common among health professionals [39], particularly because of their easy access to prescription medication. In our study, binge drinking, reduced efficiency and drinking-related absence was less common among healthcare workers than the other business areas. However, the use of psychoactive medication, both self-reported use and findings in samples of oral fluid, was higher among health professionals. Moreover, we found that use of psychoactive medication was more common among employees within transportation and storage than in other business areas.

Studies in the USA have found that in addition to hotel, restaurant and bar workers, employees within construction, building and grounds maintenance, arts, entertainment, sports and media businesses had higher odds ratios for illicit drug use and illicit drug impairment [10, 11, 32, 40] as well as working under the influence of alcohol and hangover at work [30]. An Australian study found that alcohol use at work was most common among hospitality, construction and financial services, whereas working under the influence of alcohol was most common among hospitality employees; in total, more than 5 % of the Australian workers admitted to having worked under the influence of alcohol and almost 2 % under the influence of drugs [41]. Another Australian study found that the risk of workers frequently drinking at levels associated with short-term harm was lowest in the education industry and significantly higher in the hospitality, agriculture, manufacturing and construction industries [42]. Alcohol-related absenteeism was also most common among hospitality and manufacturing employees in Australia [43]. Our study did not include the same business areas, but our findings are similar for employees within restaurant/bar and finance industries.

Finally, this study showed that the proportion who reported alcohol-related sickness absence was about twice as large as for men compared to women, a finding which is consistent with results from previous studies [8, 43], whereas reduced efficiency or absence due to drinking was reported about 60–70 % more often among men. A plausible explanation of this finding is that men drink more frequently than women and that they more often drink to intoxication than women, in all societies surveyed [44].

### Limitations

The participating employees do not represent a random selection from the total working population or the included business areas. There might have been geographical differences between urban and rural areas as well as between different parts of the country regarding the use of alcohol and drugs. Geographical areas could not be used as covariates in the statistical analysis due to low number of companies within each business area.

It is possible that some employees who had recently used alcohol or drugs refused to participate in the study because this information is regarded as sensitive. As shown above and previously [22, 34], underreporting of alcohol and drug use on the questionnaires also occurred in spite of the fact that the project team members told that the study was anonymous.

A positive drug finding in oral fluid most likely represents drug intake during the last 48 h. However, use of some drugs more than 48 h ago might also give a positive result, particularly repeated use more than a couple of days before sample collection. On the other hand, a negative oral fluid sample does not prove that drugs were not taken during the last 48 h; intake of a single dose of cannabis, cocaine or medication will in most cases cause positive oral fluid sample for less than 24 h.

One of the companies within the finance sector required that the recruitment of employees should occur in the large entrance hall. This made it possible for some employees to deliberately avoid being asked to participate; thus, the selection of participants in this company might have been somewhat biased. For all other companies, it was not possible to avoid being asked for participation.

## Conclusions

Overall, a small proportion of employees were positive for alcohol or drugs in samples of oral fluid but a significant proportion of the employees reported absence or hangover at work due to drinking. Of the studies business areas, restaurant/bar workers most often reported frequent binge drinking, reduced efficiency or absence from work due to drinking. Many of them also reported use of illicit drugs. Thus, the restaurant workers comprise a high-risk group regarding alcohol and drug use. Employees within the finance industry often reported reduced efficiency or absence because of drinking. Larger proportions of male than female workers reported binge-drinking each month, reduced efficiency or hangover at work or absence from work due to drinking during the past 12 months.
